# Look to the stars—Is there anything that public health and rehabilitation can learn from elite sports?

**DOI:** 10.3389/fspor.2022.1072154

**Published:** 2023-01-23

**Authors:** Grégoire P. Millet, Karim Chamari

**Affiliations:** ^1^ Institute of Sport Sciences, University of Lausanne, Lausanne, Switzerland; ^2^ Aspetar, Orthopedic and Sports Medicine Hospital, FIFA Medical Center of Excellence, Doha, Qatar

**Keywords:** elite sport, exercise, health, physical activity, prevention, sport and exercise medicine, therapeutic strategies.

## Introduction

Recently, several medical doctors (1) or sports scientists (2) argued that the expertise gained in elite sport should be translated and applied for the benefit of many. In other words, public health and rehabilitation could improve preventive or therapeutic methods based on exercise by looking up at what the elite athletes are doing nowadays in terms of (i) how they train and monitor adaptations to exercise; (ii) the innovation they use in combining different stressors and/or in the multi-dimensional criteria assessment of their performance.

The examples supporting this claim were however limited to “11 for health programme” (1) or to four areas (e.g., individualized training prescription; health wearables; environmental conditions; and concurrent endurance-strength training) (2). There are obviously large differences in both characteristics of the individuals and training loads undergone between elite athletes and clinical or sedentary/under-active populations Therefore, careful adaptations of any method are needed and this top-to-bottom approach may not be applied for all. However, in the present opinion paper, we suggest that the benefits of looking up at elite sports, may bring wider potential benefits than previously described. We indeed argue that there are—at least—22 topics where public health and rehabilitation can learn and benefit from knowledge and know-how started and/or developed in elite sports. In this opinion letter, we state that improved strategies leading to larger positive outcomes in clinical setting may be obtained in the following areas:

A / Exercise prescription (1. Submaximal cardiovascular assessment; 2. Individualization of exercise intensity; 3. Prescribed interval-training based on VO_2_ kinetics; 4. Submaximal strength assessment; 5. Non-invasive assessment of diffusive and convective components of O_2_ transport; 6. Non-invasive assessment of the muscle oxidative capacity; 7. Non-invasive assessment of the muscle structural properties).

B/ Monitoring of fatigue and adaptations (8. Multidisciplinary approach; 9. Specific exercise wearables; 10. Non-invasive cardiovascular monitoring; 11. Coping strategy from extreme sports).

C/ Optimizing exercise dose-response to physical activity (12. Integration of environmental conditions; 13. Concurrent endurance-strength training; 14. Weight-reduced load; 15. Vascular occlusion; 16. Neuromuscular electrical stimulation; 17. Transcranial direct current stimulation; 18. Specific responses in women; 19. Exercise training quantification; 20. Predictive models; 21. Technological innovations in mobility; 22. Ethical considerations).

This opinion letter is not aiming at presenting in detail each of these points but rather overview the areas that are—in our view—the most promising ones for a more effective application of exercise for rehabilitation and health promotion in the general public (3):

## Exercise prescription

### Submaximal cardiovascular assessment

For long, prescription of exercise intensity was mainly based on expressing exercise intensity relative to maximal values measured either during an incremental test to exhaustion [e.g., % maximal heart rate—HR—or maximal oxygen consumption—VO_2max_ (4)]. The determination of the second ventilatory threshold has later also been shown as important (5). Since the widely dissemination of the polarized training strategy in endurance athletes (6), the determination of the “aerobic” threshold (7) delineating the moderate intensity zone (8) became paramount.

The individualization of cardiovascular training based on intensity zones (e.g., moderate, heavy, severe, supramaximal) is now applied across all fitness levels, as well as being in patients recovering from cancer (9) or with musculoskeletal disorders such as low back pain (10) or osteoarthritis (11). Since there is no need of maximal exercise to determine submaximal thresholds, the accurate prescription of exercise intensity is accessible to a wider range of patients (e.g., heart failure or obese patients) for whom maximal test would be dangerous or squarely impossible (12). Moreover, digital tools are now available and render user-friendly and more accurate the non-invasive thresholds determination (13). Some authors have also suggested alternatives as the use of the walk-to-run transition speed, allowing to consider training in almost all patients, as far as they can walk fast enough to reach their “natural walk-to-run transition speed” as experimented in obese patients (14).

### Individualization of exercise intensity

In the supramaximal (i.e., above velocity associated with VO_2max_) as well as in submaximal intensity zones, exercise intensity may be prescribed based on the different thresholds. As an example, to ensure exercise is performed at low to moderate intensity, the most obvious way is to expressed intensity in %vVT1 (e.g., 90% vVT1, velocity associated with the first ventilatory threshold); or, alternatively, to ensure an exercise at higher intensity (between vVT2 and vVO2max), potentially also expressing the intensity as % within this zone (e.g., Δ50% meaning 50% of the differences between vVT2 and vVO2max). Similarly, supramaximal exercise intensity can be determined as % of anaerobic speed reserve (i.e., difference between the maximal sprint speed and vVO2max) (15). Expressing intensities in % of a given boundary (e.g., VT2) does not take into account that the other boundaries (e.g., vVO_2max_) may differ between individuals (16).

To our knowledge, exercise intensities remain commonly expressed with absolute values (e.g., km/h or W) or as % of one “marker” (e.g., %vVO_2max_) but not as % of a given intensity zone. Since patients differ to a large extent in these markers, there is a high risk of error in the prescribed intensity if expressed relative to VO_2max_, for instance (see below). These commonly used practices of prescribing exercise don't acknowledge the individual variability of these metabolic boundaries (17). This is paramount for moderate intensity that is often recommended for many patients. For instance, 60%–70% of VO_2max_ which is assumed to be a moderate exercise intensity for a fit person may in fact corresponds to supra-VT1, i.e., “heavy” intensity for some individuals. This would induce delayed post-exercise autonomic recovery and increased risk of overreaching or overtraining (18).

### Prescribed interval-training based on VO_2_ kinetics

The “traditional” determination of the exercise intensity zones by the ventilatory or lactic thresholds has been challenged by a relatively recent approach based on the VO_2_ kinetics (19). That is the profile of VO_2_ following the onset of constant-load exercise that defines the exercise-intensity domain (e.g., moderate, heavy, severe, supramaximal) (8) in which the exercise is performed. The individualization of training based on these intensity zones is now applied across all fitness levels (19). The analysis of the fast and slow components of the VO_2_ dynamic response to exercise is paramount in endurance sports (8). Physiological responses to sessions of interval-training (20) or repeated sprints (21) are related to the VO_2_ kinetics parameters of the individuals. Moreover, the effectiveness of these sessions is increased by adjusting work-interval duration or intensity based on the VO_2_ kinetics parameters. For example, athletes with a slow VO_2_ kinetics would benefit to a large extent of increasing the duration of the work-interval (e.g., 30 s to 60 s) (20) or increasing the work-interval intensity (e.g., from 100% to 105% vVO_2max_) (22). The investigation of VO_2_ kinetics is performed in several diseases (19) as chronic obstructive pulmonary disease (23), type-2 diabetes mellitus (24) or obesity (25). An important consideration is that this method does not require the patient to perform intense exercise to be assessed. It is beyond the scope of this article to describe how VO_2_ kinetics is altered across these diseases [see (19)] but to our knowledge there is no report yet of exercise prescription taking into account this factor in clinical populations.

### Submaximal strength assessment

Determination of the load for strength training is commonly based on a maximal strength test (e.g., expressing the load in % of 1 RM, one repetition maximum (26). Since accurate loads can even be prescribed based on submaximal assessments (e.g., 6–12 RM) (27), strength training is henceforth relying less on % 1-RM. The possibility to estimate 1RM through submaximal tests (e.g., 6RM) to minimize the risk of injury would enable many patients (e.g., with osteoarthritis) (28) to benefit from accurate load prescription from this type of submaximal strength assessment for their muscle strengthening programs, hopefully bringing several supplementary beneficial health effects.

An alternative strength training method consists in velocity-based resistance training now commonly used successfully by strength-power athletes (29). To our knowledge, this innovative method is less frequently used in patients or elderly individuals known to develop their force at a lower rate for eliciting a lower rate of force development (30).

### Non-invasive assessment of diffusive and convective components of O_2_ transport

VO_2max_ is determined by both convective and diffusive limitations, as described by (31). This model requires invasive collection of arterial and muscle venous blood. Near-infrared spectroscopy (NIRS) was recently proposed as a non-invasive approach to investigate these components. NIRS-derived signals of tissue saturation (Tissue Saturation Index, TSI) reflects the balance between O_2_ delivery and O_2_ utilization at the microvascular level within working skeletal muscles. This is allowing to non-invasively estimate the convection and diffusion components of VO_2max_ (32). This promising method could optimize the services in clinical populations (33).

### Non-invasive assessment of the muscle oxidative capacity

The muscle oxidative capacity is assessed from muscle tissues collected by invasive biopsies (34). This technique enables to investigate the mitochondrial adaptations (respiration, biogenesis …) following a given training intervention (35). NIRS is a method that has been used to determine the changes in semi-superficial muscle oxygenation in athletes (36) following various training intervention as strength (37) or hypoxic training (38). Recently, NIRS has been shown as a valid and non-invasive means of assessing both muscle oxidative and oxygen diffusing capacity *in vivo* (39). Such monitoring of both cardiovascular fitness and muscle oxidative properties may be valuable in many at-risk individuals; e.g., patients with obesity (40). Moreover, through exercise-induced myokines and muscle-to-brain signaling pathways (41), this non-invasive assessment could be used beyond cardiovascular or metabolic dysfunction; e.g., for patients with neurodegenerative diseases, as Parkinson disease (42).

### Non-invasive assessment of the muscle structural properties

The determination of the muscle properties requests invasive biopsies to determine muscle structure, fibres composition as well as adaptation to a given exercise (e.g., muscle damage) (43). Histological analysis enables an accurate determination of the fast- vs. slow- twitch fibres composition (44). Recently a non-invasive alternative based on the muscle carnosine content by Proton magnetic resonance spectroscopy (45) has been developed with direct application in elite sport (46). Such innovation in the determination of human muscle fibre type composition is promising for many neuromuscular diseases' patients (e.g., myopathy) (47).

### Combination of biomechanical, physiological and psychological parameters

Sport scientists and coaches aim to assess or improve the energetics, biomechanics and mental characteristics of their elite athletes. Training in endurance sports consists in increasing the total energy available (i.e., increasing the maximal aerobic power and the anaerobic capacity), decreasing the energy expenditure at a given intensity by improving the locomotion mechanics/technics of the athletes (48). Variables combining energy expenditure and velocity [e.g., energy cost (49)] or power output efficiency (50)) are used to determine the level or training adaptations in athletes. Similarly, multi-criteria psycho-physiological scales (e.g., combining objective training intensity/load and subjective feeling/mood responses) are relevant for monitoring athletes. Despite some limitations, rating of perceived exertion [Borg's scale (51)] is probably one of the most used tool in sport sciences. One of the main application of RPE is to quantify in a very simple way, the training load undergone by athletes to prevent overreaching (52). The integration of multidisciplinary methods has been shown to be highly relevant in elite athletes and to our knowledge is less spread in the clinical setting, certainly warranting more research in the field.

## Monitoring of fatigue and adaptations

### Specific exercise wearables

Until 20 years ago, sport science was limited to laboratory setting/testing. We needed non-wearable equipment to measure gas exchange, muscle oxygenation or muscle electromyography activity. For long, only HR was measured with HR monitors. Thanks to technological advancement and miniaturization of the devices, it is now possible to measure several time motion and/or physiological responses during exercise in the field [e.g., speed (GPS); power output (powermeter); stride frequency and several running mechanics parameters as contact time (Inertial Measurement Units); forces in the pedals or under the running shoes; heart rate (HR and HR variability); muscle oxygenation (portable Near-infrared spectroscopy); blood pressure; arterial saturation (pulse oximeter); breathing frequency (instrumented shirts); glycaemia by CGM (continuous glucose monitor); central temperature (ingestible pills); galvanic skin response; thoracic impedance (impedance sensors);… ]. Of importance is that many of these exercise wearables are now certified by the medical authorities (e.g., Apple Watch for ECG function approved by the FDA in USA). Sport is an area where there are many technological innovations and where many wearables are used. Not all the devices mentioned above are accessible for a daily use for the active individuals in the community but some have changed the way people train at all levels [e.g., GPS in running (53); powermeter in cycling (50)]. A key issue is about the accuracy and reliability of these wearables that are highly promoted by the industry (54). In fact, due to remaining limitations in data storage and algorithmic computation, very few of these devices are relevant for constant 24 h/24 h ambulatory monitoring of patients. In clinical setting, wearables are mainly used for safety reasons as surveillance and monitoring of the individuals. We claim that many wearables would also be useful to motivate and to monitor changes within the individual progress of the patients, and to provide continuous feedback for a more accurate and efficient exercise prescription (55, 56).

### Non-invasive cardiovascular monitoring

Preventing overreaching and overtraining is an important concern for coaches and sport scientists (57). Among various means, resting heart rate variability or photoplethysmography (58) have been used in elite performance area, particularly in endurance sports. Despite that there is no consensus about the optimal procedures or variables to be measured, HRV is recognized as effective and is recorded on a daily basis by numerous elite athletes (59, 60).

HRV is also used extensively in clinical setting with many different types of patients (61, 62). HRV is then seen mainly as a valuable diagnostic metrics of health. However, HRV is rarely used for guiding, monitoring or amending a rehabilitation program as it is performed in sport where training program can be HRV-guided (63). Similarly, the monitoring of HR recovery post-exercise may be a complementary variable for monitoring the progress during rehabilitation, as shown in patients after acute coronary syndrome (64).

### Coping strategy coming from extreme sports

Ultramarathons, with specific neuromuscular, renal, inflammatory and cardiovascular alteration/recovery kinetics, have been proposed as a unique experimental model to explore the responses of healthy humans to extreme load, fatigue and stress (65). Among those complications, three “specific” diseases may lead the patient to intensive care units (ICU): malignant hyperthermia, rhabdomyolysis or hyponatremia. However, contrary to many patients admitted in ICUs who are often with comorbidities, ultra-endurance runners are healthy individuals, which often makes the treatment or investigation of the mechanisms associated to such diseases easier than in the clinical population. This may suggest that clinicians would benefit from the reports and observations from ultra-endurance athletes or sport scientists involved in this field. For example, it is possible to investigate regulatory mechanisms or organs interplay as the relationship between acute kidney injury and some adverse cardiac dysfunctions (66). Another example comes from professional cycling: arterial endofibrosis is a common pathology in cyclists (67) and it led to clinical improvement in its diagnosis and treatment (68).

## Dose-Response to exercise

### Integration of environmental conditions as heat, cold or hypoxia

Altitude/hypoxic training (69) and heat acclimatization (70) are very important components of preparation in elite sport. Recent innovative methods have been developed [e.g., to cite only few: repeated sprint training in hypoxia—RSH—for team sport athletes (71); warm bath (72) for heat acclimatization); full body cryotherapy for improving recovery or rehabilitation (73)…]. Combination of several environmental stressors is also investigated (74). Some mechanisms explored in sport lead to clinical advancements [e.g., RSH-induced compensatory vasodilation that may be of interest in angiology for treatment of patients with endothelial dysfunction (75)]. Intermittent hypoxic conditioning, have been implemented for general health purposes or in clinical populations, for example in patients with diabetes (76) or neurodegenerative diseases (77). The effective “dose” (e.g., hypoxia severity; temperature level, duration and intermittent pattern of exposure) for any of these conditions are likely specific to type of patient and disease (78) and certainly warrants further promising investigations.

### Concurrent endurance-strength training

For long, endurance athlete did not perform any strength training believing that this would be counterproductive. Nowadays however, strength and power training is an important component of the endurance athletes preparation (79). A large body of research has been published on the optimal combination of strength and aerobic training (i.e., concurrent training) for endurance and strength enhancement. Overall, it seems that strength-training—particularly if it includes plyometric stimuli—could be beneficial for endurance performance, while hypertrophy may be blunted by large amount of low-intensity training. The intra-session session sequence (i.e., endurance prior strength or vice-versa) is a question of importance in elite sport (80), general population (81) or in rehabilitation (82). For instance, the use of plyometric exercise has been shown to be beneficial for young obese females by reducing the pre-training observed metabolic abnormalities (83).

Similarly, the recommendations for exercise in elderly (i.e., “*healthy aging*”) are no longer limited to aerobic exercise for cardiovascular maintenance but also integrate exercises dedicated to the neuromuscular function (84); e.g., strength training for counterbalancing muscle force loss and sarcopenia (85); flexibility to maintain join amplitude or proprioception and agility/coordination to counterbalance altered balance and reduce the risks of fall (86). Strength training has indeed been shown to be effective even at very old ages (e.g., nonagenarians) (87).

### Weight-reduced modalities (low-G; hypoxia; immersion)

For injured or in-rehabilitation athletes, several methods have been developed to decrease the mechanical load and enable exercise despite biomechanical constraints or pain like: running at relative lower body weight on a Low-Gravity treadmill (88), on a water immersion treadmill (89), uphill (90) or in hypoxia (91). The knowledge acquired on the physiological responses in such conditions may be useful for many clinical populations with locomotion limitation; for example for patients who are subject to excessive body weight (92) and/or elderly patients (93).

### Vascular occlusion

Strength training at low load with vascular occlusion (e.g., blood flow restriction; BFR) enables gains in hypertrophy similar to those obtained with high-loads (94). Developed originally for clinical application, BFR is now extensively used by strength/power athletes looking for muscle mass development (95). This created an increased knowledge on the benefits and limitations of this method that is now benefiting the therapeutic applications again (96) in many diseases or pathologies. This has already been used in diabetic patients (97), for muscular rehabilitation (98), or to counterbalance sarcopenia in older individuals (99).

### Neuromuscular electrical stimulation

Since early 70 s, neuromuscular electrical stimulation (NMES) has been used for increasing muscle strength in athletes and its use has been soon transferred to clinical patients (100). Recent improvement in methods, devices and impulse characteristics has improved its effectiveness in both athletic (101), sedentary (102) or elderly (103) individuals. There is a large interest for the use of NMES in rehabilitation with many avenues for improving adaptations (104), particularly by implementing algorithm-based NMES therapy and dosing the treatment with tension-controlled NMES (105).

### Transcranial direct current stimulation

Transcranial direct current stimulation (tDCS) is one of the non-invasive brain stimulation techniques aiming at modulating cortical excitability (106). In sport, tDCS has been shown as a potential means of improving performance (107), particularly time to task failure (108). More recent meta-analyses reveal that the ergogenic effect of tDCS is probably small (109). Similarly, its therapeutic effectiveness in many pathologies (e.g., pain, Parkinson's disease, motor stroke, multiple sclerosis, epilepsy, Alzheimer's disease, depression, schizophrenia) is likely moderate (110). However, even if not supported by evidence, the recent interest for such “brain doping” techniques in sport (111, 112) may results in improvements in methods and devices, and presumably in ethical debates in a close future.

### Specific responses in women

In both sport and exercise science (113) or medicine (114) areas, there is a lack of studies on the specificities of women, that are underrepresented in science, not only as researchers but also as research participants (115). Moreover, beyond these quantitative considerations, there is a major concern regarding methodological limitations (e.g., consideration of menstrual cycle phase, type of hormonal contraceptives and hormone replacement therapy, stage of menopause) and the low quality of many studies that precludes the generalization of the results to the entire female population (113, 116). There is an increasing body of recent researches in sport sciences about specific characteristics of women; e.g., the effects of menstrual cycle (117), pregnancy (118) or menopause (119) on the responses to exercise. This led to improved management of training load (120) or reduced RED-S (i.e., relative energy deficiency in sport) (121) in elite female athletes. To our knowledge, this body of research on the prescription of exercise for preventive or therapeutic purposes is less developed, warranting promising female-centred research for the future.

### Exercise training quantification

Several methods of quantification of the training stimulus (e.g., training loads) are used in sport science. Based on the records of HR (122), training velocities (123) or RPE (124) for every training session, these training load assessment methods aim at quantifying the load either to improve sport performance and/or to prevent overreaching (52), illnesses (125) or injuries (126). Training loads are also the foundation of the different types of periodization in sport; i.e., polarized (6), block (127) or pyramidal (128) training. The translation of these training patterns, based on objective training load measurement, may benefit the long-term exercise-based rehabilitation program (129).

### Predictive models of the exercise effects on health

The relationships between training content/load and fluctuation of performance are extensively investigated in elite sport (130). Initiated by Banister (122), several model exist and may help predicting short- or long-term changes in performance (131). In most of these models, performance is calculated as the balance between negative (i.e., fatigue) and positive (i.e., fitness) antagonistic functions. Such models were shown useful in many sports as triathlon (132) or swimming (133). Such predictive models of the relationships between exercise stimulus and the primary health outcomes may also be effective in clinical setting but may require to combine different types of monitored variables beyond training load (e.g., neuromuscular function, HRV, psychological wellbeing, past injury history) similarly to an approach implemented in elite sport (134).

### Technological innovations in mobility

Paralympic elite sport is a growing area of innovation in sport sciences. Many paralympic sports require sophisticated technological modifications (e.g., in prosthetic and wheelchair devices) (135). Such technology advancement may be seen as unfair (136) and the potential technological advantage for a Paralympian, when competing against an Olympian, is unclear (137). However, beyond performance, the innovation is also for improving safety (138) with a direct impact for the stability/safety of equipment used daily by disable individuals (139), potentially bringing to the latter a substantial improvement of their quality of life.

### Ethical considerations

The increase of data availability has potential associated issues, as data ownership and data transfer could potentially lead to ethical issues in case of breach of confidentiality. In elite sport, a recent gathering of professional football (soccer) players can give glimpse on the potential issues with the ownership and use of the data (https://fifpro.org). The latter group of players and professionals created the Charter of Player Data Rights, aiming at implementing global industry standards in order to protect the players' privacy and allowing them to protect and benefit from personal rights for the access and management of the information relative to their health status and performance scores. We believe that the developers of health care technologies and tools shall continuously check the data protection rules/laws, in order to be proactive in this field hopefully ensuring a healthy use of their products by respecting ethics and data confidentiality (EU's 2016 General Data Protection Regulation GDPR).

## Conclusion

In this opinion letter, we listed 22 topics where sport sciences may benefit and be of great value for enhancing public health and rehabilitation. We acknowledge that there may be other points where a better interplay between sport scientists and medical researchers should be valuably considered. To conclude, the main objective of this letter is to claim for a better integration of exercise science—including the ones that has been developed with elite athletes—for a greater use in preventive medicine for a better health in the general population.
