# Erratum: Look to the stars—Is there anything that public health and rehabilitation can learn from elite sports?

**DOI:** 10.3389/fspor.2023.1170304

**Published:** 2023-03-17

**Authors:** Frontiers Production Office

**Affiliations:** Frontiers Media SA, Lausanne, Switzerland

**Keywords:** elite sport, exercise, health, physical activity, prevention, sport and exercise medicine, therapeutic strategies

An Erratum on Look to the stars—Is there anything that public health and rehabilitation can learn from elite sports? By Millet GP and Chamari K. (2023) Front. Sports Act. Living. 4:1072154. doi: 10.3389/fspor.2022.1072154

An omission to the funding section of the original article was made in error. The following sentence has been added: “Open access funding was provided by the University of Lausanne”.

The original version of this article has been updated.

